# Clinical aspects and short-term prognosis in a cohort of patients with infective endocarditis, São Paulo, Brazil

**DOI:** 10.1590/1980-220X-REEUSP-2025-0060en

**Published:** 2025-07-28

**Authors:** Juliana Barros Becker, Valdir Ambrósio Moisés, Dulce Aparecida Barbosa

**Affiliations:** 1 Universidade Federal de São Paulo, Hospital Universitário, São Paulo, SP, Brazil.; 2 Universidade Federal de São Paulo, Escola Paulista de Medicina, São Paulo, SP, Brazil.; 3 Universidade Federal de São Paulo, Escola Paulista de Enfermagem, Departamento de Enfermagem Clínica e Cirúrgica, São Paulo, SP, Brazil.

**Keywords:** Endocarditis, Cross Infection, Epidemiology, Hospital Mortality, Staphylococcus, Methicillin-Resistant Staphylococcus aureus, Endocardite, Infecção Hospitalar, Epidemiologia, Mortalidade Hospitalar, Staphylococcus, Staphylococcus aureus Resistente à Meticilina

## Abstract

**Objective:**

To analyze the clinical characteristics of patients with infective endocarditis (IE) admitted to a tertiary hospital in Brazil, in-hospital mortality and predictors of readmission and mortality up to six months after hospital discharge.

**Method:**

A retrospective cohort study, with data collected from medical records of patients with diagnosis of IE hospitalized during the study period. For comparative statistical analysis, patients were grouped according to survival and death outcomes.

**Results:**

A total of 204 patients participated in the study. Healthcare-associated IE accounted for 62.3% of cases, with *Staphylococcus aureus* as the predominant pathogen. Mortality was significantly associated with complications such heart failure and septic shock (p < 0.001). Diabetes mellitus (OR 7.76; p < 0.001) and acute kidney injury (OR 7.99; p = 0.016) were independent risk factors for hospital readmission. Overall mortality was 50.9%.

**Conclusion:**

Short-term mortality was high. Healthcare-associated infections were predominant, and complications and comorbidities significantly affect mortality in IE patients. Identifying high-risk patients and optimizing management may improve outcomes.

## RESUMO

**Objetivo:**

Analisar as características clínicas de pacientes com endocardite infecciosa (EI) admitidos em um hospital terciário no Brasil, a mortalidade intra-hospitalar, preditores de readmissão e mortalidade até seis meses após a alta.

**Método:**

Estudo de coorte retrospectivo, com coleta de dados de prontuários de pacientes com diagnóstico de EI hospitalizados durante o período do estudo. Para a análise estatística comparativa, os pacientes foram agrupados conforme os desfechos sobrevida e óbito.

**Resultados:**

204 pacientes participaram do estudo. EI associada à assistência à saúde foi responsável por 62,3% dos casos, e Staphylococcus aureus foi o patógeno predominante. A mortalidade foi significativamente associada a complicações como insuficiência cardíaca e choque séptico (p < 0,001). Diabetes mellitus (OR 7,76; p < 0,001) e lesão renal aguda (OR 7,99; p = 0,016) foram fatores de risco independentes para readmissão hospitalar. A mortalidade total foi de 50.9%.

**Conclusão:**

A mortalidade de curto prazo foi elevada. Infecções associadas à assistência foram predominantes, e comorbidades e complicações afetaram significativamente a mortalidade em pacientes com EI. A identificação de pacientes de alto risco e a otimização da gestão de cuidados podem melhorar os desfechos.

## INTRODUCTION

Infective endocarditis (IE) is defined by an infection of the endocardial surface of the heart, a native or prosthetic heart valve or an indwelling cardiac device. It is a disease of global magnitude that remains with high mortality, approaching 30% in one year and significant morbidity, including prolonged hospital stay, reduced quality of life and an elevated risk of re-infection. Despite advances, incidence rates have increased in recent years. In high-income countries, this increase can be explained by changes in the epidemiological profile, such as population aging, greater exposure to healthcare procedures, increased intracardiac and vascular device use, and the emergence of *Staphylococcus* infections^(1,2)^.

In relation to Latin America, data from the “Global Burden of Disease Study” indicate that the region had the highest incidence rates of IE, and Brazil was the country with the greatest global increase in IE incidence rates in 2019^(3)^. Recent local studies show in-hospital mortality between 32% and 50%^(4,5,6,7,8,9)^, placing Brazil as one of the countries with the highest risk of death from IE.

Healthcare-associated infective endocarditis (HAIE) currently has a prominent place in the epidemiology of the disease, accounting for 25% to 35% of IE cases in contemporary cohorts in high-income countries and 35% to 67% of cases in national cohorts^(2,4,6)^. The most recent guideline on IE management published by the European Society of Cardiology emphasizes the importance of the integrated action of the multidisciplinary team in prevention and health education measures for patients at increased risk of IE ^(10)^.

The scenario is challenging and, despite the alarming data, changes in the epidemiological and microbiological profile of patients with IE in Brazil are still poorly known. Some data from the research, such as differences according to IE classification and predictors for in-hospital mortality, were recently published^(11)^. This present study aimed to analyze the clinical characteristics of patients with IE admitted to a tertiary hospital in Brazil, in-hospital mortality and predictors of readmission and mortality up to six months after hospital discharge.

## METHOD

### Study Design and Setting

A retrospective cohort study was carried out between 2020 and 2023, with a data collection period from January 2009 to December 2019, at the *Hospital São Paulo*, *Universidade Federal de São Paulo*, Brazil. The hospital is a reference in care, teaching, and research in Brazil.

### Participants

Patients with more than 18 years old hospitalized in the period analyzed with suspected IE were initially selected from the echocardiography laboratory database. Patients were finally included in the study after a diagnosis of confirmed or possible IE according to modified Duke criteria^(12)^ and if all data were available in the physical (2009 to 2015) and electronic (2016 to 2019) medical records. Patients were divided and compared into two groups according to survival or death outcomes. Patients who survived and continued follow-up were analyzed, including hospital readmissions and deaths within six months after discharge.

### Data Collection

The data collected included demographic characteristics, predisposing cardiac condition for IE, comorbidities, causative microorganisms, mode of acquiring IE, clinical and echocardiographic findings, complications, and surgical treatment. For patients admitted to the Intensive Care Unit (ICU) for more than 48 hours, Acute Physiology and Chronic Health Evaluation (APACHE II) and Sequential Organ Failure Assessment (SOFA) scores were calculated within the first 24 hours^(13,14)^.

### Variable Definitions

Predisposing cardiac condition was defined as a history of heart valve replacement, congenital heart defects, valve disease, previous episode of IE, and cardiac device implantation such as a pacemaker or defibrillator. IE complications included acute heart failure, cardiogenic shock, arterial embolisms including pulmonary infarction, sepsis, septic shock, and acute kidney injury (AKI) requiring hemodialysis. Cardiogenic shock was defined according to stages C, D and E of the Society for Cardiovascular Angiography and Intervention^(15)^. Sepsis was defined as organ dysfunction caused by a dysregulated response to infection. Septic shock was defined as sepsis with resistant hypotension that requires vasopressors to maintain mean arterial pressure ≥ 65 mmHg^(16)^. AKI was defined according to Kidney Disease: Improving Global Outcomes (KDIGO) criteria^(17)^. The causative agents of IE were divided according to the resistance pattern into susceptible, multidrug-resistant (MDR), extensively drug-resistant (XDR), and pandrug-resistant (PDR). MDR was defined as acquired non-susceptibility to at least one agent in three or more antimicrobial categories; XDR was defined as non-susceptibility to at least one agent in all but two or fewer antimicrobial categories; and PDR was defined as non-susceptibility to all agents in all antimicrobial categories^(18)^.

The modes of acquisition of IE were HAIE or community- acquired infective endocarditis (CIE). HAIE was divided into non-nosocomial and nosocomial. Non-nosocomial IE was defined as occurring before or within 48 hours of hospital admission in a patient with extensive out-of-hospital exposure to medical interventions; hospital admission for two or more days within 90 days before onset of IE; residence in a nursing home or long-term care facility prior to hospital admission. Nosocomial IE was defined as those whose signs and symptoms compatible with IE developed after 48 hours or more of hospital admission. CIE was defined as IE whose signs and symptoms developed before or within 48 hours of hospital admission and that did not meet the criteria for non-nosocomial IE^(19)^.

### Statistical Analysis

The collected data were analyzed using the free R version 4.2.2 for Windows software. In descriptive data analysis, quantitative variables are summarized through mean, standard deviation, median, interquartile range according to the type of distribution; qualitative variables are presented as numbers and percentages. Likewise, comparative statistical analyzes between groups were performed using the chi-square test or Fisher’s exact test for qualitative variables. For quantitative variables, Student’s t test or Mann-Whitney test were used. Multivariate logistic regression was performed to identify variables related to hospital readmission and mortality after discharge, and the Odds Ratio was calculated with a 95% Confidence Interval. P-values less than 0.05 were considered statistically significant.

### Ethical Aspects

The study was approved by the *Universidade Federal de São Paulo* Research Ethics Committee (Resolution 673/2019, Opinion 3,588,513).

## RESULTS

The initial sample consisted of 278 patients with suspected IE; of these, 204 met the inclusion criteria. Table 1 summarizes the main clinical characteristics. Men accounted for 57.8% of this cohort, with no differences between outcomes. The mean age was 53 years, with a higher mean in patients in the death outcome (p < 0.001). History of ischemic heart disease (p = 0.003) and hypertension (p = 0.026) was observed in a higher percentage in the death outcome. Regarding the survival outcome, a higher percentage of patients with a history of recent bloodstream infection (up to 90 days) was observed (p = 0.019). Concerning symptoms, fever was observed in 88.7% of patients, and heart murmur was heard in 52.5%, with no difference between groups.

**Table 1:** Characteristics of patients with infective endocarditis according to outcomes – São Paulo, SP, Brazil, 2009–2019.

	Survival (n = 113)	Death (n = 91)	Total (n = 204)	p-value
Male	64 (56.6)	54 (59.3)	118 (57.8)	0.697
Median age (SD)	47.45 (16.779)	60.11 (14.373)	53.21 (16.887)	<0.001
Predisposing cardiac conditions				
Previous valvular disease	18 (15.9)	23 (25.3)	41 (20)	0.097
Rheumatic heart disease	11 (9.7)	8 (8.8)	19 (9.3)	0.817
Previous IE episode	10 (8.8)	9 (9.9)	19 (9.3)	0.799
Cardiac device	6 (5.3)	8 (8.8)	14 (6.9)	0.328
Previous cardiac surgery				
Coronary artery bypass grafting	2 (1.8)	5 (5.5)	7 (3.4)	0.246
Valve surgery	14 (12.4)	16 (17.6)	30 (14.7)	0.298
Others	8 (7)	6 (6.6)	14 (6.9)	0.891
Comorbidities				
Ischemic heart disease	10 (8.8)	22 (24.2)	32 (15.7)	0.003
Heart failure	23 (20.4)	21 (23)	44 (21.6)	0.638
Other cardiopathies	18 (15.9)	18 (19.8)	36 (17.6)	0.473
High blood pressure	65 (57.5)	66 (72.5)	131 (64.2)	0.026
Cancer	14 (12.4)	12 (13.2)	27 (13.2)	0.817
Diabetes	28 (24.8)	28 (30.7)	56 (27.5)	0.341
Hemodialysis	42 (37.2)	35 (38.5)	77 (37.7)	0.847
Intravascular catheter (central)	39 (34.5)	27 (29.7)	66 (32.4)	0.462
Recent blood stream infection	14 (12.4)	3 (3.3)	17 (8.3)	0.019
Recent hospital admission	30 (26.5)	27 (29.7)	57 (27.9)	0.621
Prosthetic valve endocarditis	12 (10.6)	14 (15.4)	26 (12.7)	0.310
CIE	43 (38)	34 (37.4)	77 (37.7)	0.919
Non-nosocomial HAIE	51 (45.1)	32 (35.2)	83 (40.7)	0.149
Nosocomial HAIE	19 (16.8)	25 (27.5)	44 (21.6)	0.066
Clinical findings at presentation				
Fever	102 (90.3)	79 (86.8)	181 (88.7)	0.438
Heart murmur	56 (49.6)	51 (56)	107 (52.5)	0.356
Duke criteria				
Major				
Positive echocardiogram	84 (74.3)	87 (95.6)	171 (83.8)	<0.001
Positive blood culture	47 (41.6)	43 (47.3)	90 (44.2)	0.418
Minor				
Predisposing heart condition	41 (36.3)	45 (49.5)	86 (42.2)	0.058
Central venous catheter use	44 (38.9)	21 (23)	65 (31.8)	0.016
Vascular phenomena	41 (36.3)	44 (48.4)	85 (41.7)	0.082
Immunologic phenomena	19 (16.8)	18 (19.8)	37 (18.2)	0.585
Blood culture (not major)	25 (22.2)	3 (3.3)	28 (13.7)	<0.001
IE				
Possible	41 (36.3)	12 (13.2)	53 (26)	<0.001
Definitive	72 (63.7)	79 (86.8)	151 (74)	<0.001

Note: p-value = chi-square test or Fisher’s exact test for qualitative variables and Student’s t test for quantitative variables; SD – standard deviation; CIE = community-acquired infective endocarditis; HAIE = healthcare-associated infective endocarditis; IE = infective endocarditis.

Definitive IE was diagnosed in 151 patients (74%) with significantly higher proportion in the death outcome group (p < 0.0001) and possible IE in 53 patients (26%) with higher proportion in the survival outcome group. HAIE accounted for 62.3% of cases in this cohort; 40.7% were classified as non- nosocomial, and 21.6% as nosocomial, without significant difference between groups.

Transesophageal echocardiography was performed on all patients. The mitral valve was the most affected by vegetation, especially in the death outcome (p = 0.026), followed by the aortic valve, with no differences between groups. In the death outcome, a greater number of vegetations larger than 10 mm (p = 0.003) and complications, such as leaflet perforation, pseudoaneurysm and perivalvular abscess (p = 0.016), were observed.

**Figure 1:**
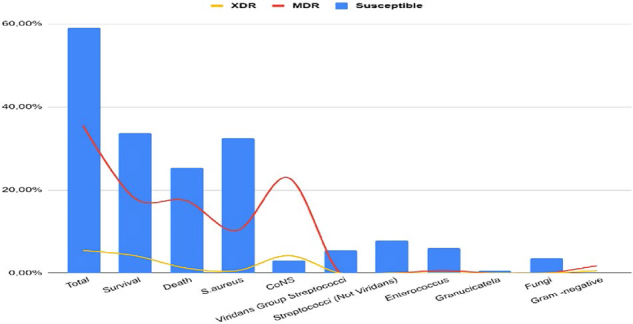
Causative infective endocarditis agents according to resistance profile.

Note: MDR – multidrug resistant; XDR – extensively drug-resistant.

The blood cultures were negative in 18.1% cases, without significant difference between groups. Among the positive blood cultures, gram-positive bacteria were found in 77% of cases, of which 45.9% were *Staphylococcus aureus* and 31.8% were coagulase-negative *Staphylococcus* (CoNS). Around 26% of *Staphylococcus aureus* and 84% of CoNS samples showed resistance to methicillin, with no significant difference between groups. Resistance classified as MDR was observed in 35% and as XDR in 5% (Figure 1). Endocardial or valve tissue cultures were performed in forty patients (57.1%) undergoing surgical treatment. Of these, 85% did not show microbial growth, with no difference between outcomes. There were no changes in international guidelines on antimicrobial therapy for IE during study period^(2)^. The pharmacological treatment used was b-lactam antimicrobial agents in 91%, glycopeptides in 84%, with no significant difference between groups. Among patients with death outcome, there was greater use of aminoglycosides (p = 0.027), polypeptides (p < 0.001) and antifungal agents (p = 0.004) during hospital stay.

Patients with death outcome presented the majority of complications. Embolic events occurred in around 60% of patients in this group (p = 0.002), and the central nervous system was the most affected (p < 0.001). AKI requiring hemodialysis was observed in 47% (p < 0.001) of these patients. Healthcare-associated infections were also more frequently observed in patients with this outcome: 57.1% contracted bloodstream infection and 58.2% pneumonia (p < 0.001). More than 60% of patients with death outcome developed septic shock (p < 0.001); 48% developed heart failure (p < 0.001); 25% developed cardiogenic shock (p < 0.001); 29.7% developed cardiorespiratory arrest (p < 0.001); 83% required invasive mechanical ventilation (p < 0.001); and 94.5% needed ICU admission (p < 0.001). Patients with the death outcome also had higher scores on APACHE II (p = 0.001) and SOFA (p = 0.002) scores. The mean length of hospital stay was 44.67 days, with a higher mean for survival (p = 0.016) (Table 2).

**Table 2:** Complications observed during hospital admission of patients with infective endocarditis according to outcomes – São Paulo, SP, Brazil, 2009–2019.

	Survival (n = 113)	Death (n = 91)	Total (n = 204)	p-value
Embolic events	41 (36.3)	53 (58.2)	94 (46.1)	0.002
Central nervous system	14 (12.4)	34 (37.4)	48 (23.5)	<0.001
Pulmonary	10 (8.8)	2 (2.2)	12 (5.9)	0.045
Osteoarticular	4 (3.5)	6 (6.6)	10 (4.9)	0.346
Others	13 (11.5)	11 (12.1)	24 (11.8)	0.897
Infectious complications				
Sepsis	28 (24.8)	22 (24.17)	50 (24.5)	0.921
Septic shock	17 (15)	58 (63.7)	75 (36.8)	<0.001
Healthcare-associated infections				
Bloodstream infection	36 (31.8)	52 (57.1)	88 (43.1)	<0.001
Pneumonia	28 (24.8)	53 (58.2)	81 (39.7)	<0.001
Urinary tract infection	15 (13.3)	18 (19.8)	33 (16.2)	0.209
Others	14 (12.4)	6 (6.6)	20 (9.8)	0.166
Heart failure	17 (15)	44 (48.4)	61 (29.9)	<0.001
Cardiogenic shock	1 (0.9)	23 (25.3)	24 (11.8)	<0.001
Cardiorespiratory arrest	3 (2.7)	27 (29.7)	30 (14.7)	<0.001
AKI				
AKI without hemodialysis	28 (24.8)	17 (18.7)	45 (22.1)	0.296
AKI with hemodialysis	15 (13.3)	43 (47.2)	58 (28.4)	<0.001
Transfusion of packed red blood cells	39 (34.5)	55 (60.4)	94 (46.1)	<0.001
Invasive mechanical ventilation	32 (28.3)	76 (83.5)	108 (52.9)	<0.001
ICU	71 (62.8)	86 (94.5)	157 (76.9)	<0.001
Severity ICU scores^|^|				
Apache II (Mean – SD)	16.4 (9.9)	22.24 (8.75)	19.5 (9.72)	<0.001
0 to 14 points (15% risk of death)	28 (40.6)	7 (8.8)	35 (23.5)	<0.001
15 to 29 points (25–55% risk of death)	37 (53.6)	55 (68.8)	92 (61.7)	0.058
30 to > 34 points (73–85% risk of death)	4 (5.8)	18 (22.5)	22 (14.8)	0.004
SOFA	6.61 (3.61)	8.419 (3.998)	7.591 (3.992)	0.002
0 to 5 points (20.2% risk of death)	28 (40.6)	15 (18.3)	43 (28.5)	0.002
6 to 11 points (50% risk of death)	37 (53.6)	51 (62.2)	88 (58.2)	0.287
>12 points (95.2% risk of death)	4 (5.8)	16 (19.5)	20 (13.2)	0.013
Length of stay in ICU	14.45 (13.416)	22.5 (25.053)	18 (20.877)	0.075
Length of hospital stay	45.5 (21.57)	43.65 (36.3)	44.67 (29.03)	0.016

Note: AKI – Acute Kidney Injury; ICU – Intensive Care Unit; p-value = chi-square test or Fisher’s exact test for qualitative variables and Mann-Whitney test for quantitative variables; ^||^Severity ICU scores: calculated on 149 patients; APACHE II = Acute Physiology and Chronic Health Evaluation score; SD = standard deviation; SOFA = Sequential Organ Failure Assessment score.

There was an indication for surgery for 42.6% of patients in this cohort, and 34.3% of patients underwent surgical treatment. Of the patients who had indication for surgery, almost 20% did not undergo surgery, mainly due to the death outcome (p = 0.024). The mean time between diagnosis of IE and surgical procedure was 22 days. Valve replacement surgery with biologic prosthesis placement was performed in 56% of cases. Mitral (44.3%) and aortic (21.4%) valves were the most affected, with no differences between outcomes.

Of the 204 patients in this cohort, 91 (44.6%) died during hospital stay. Among the 113 patients who survived, 82 (72.6%) maintained follow-up at the institution in the six months following discharge. Of these, 30 patients (36.6%) required new hospital admission in this period, and 13 more patients died. So, since the primary hospital admission and considering patients under follow-up, a total of 104 patients died.

The main causes of hospital readmission were sepsis (56.7%) and heart failure (20%). Bloodstream infection and pneumonia were the main causes of infection. IE recurrence accounted for 10% of cases. Patients that required new hospital admission had higher mean age (50 years old; p = 0.04) and higher percentage of hypertension (76.6%) (p = 0.003), diabetes mellitus (46.7%) and hemodialysis (63.3%) (p < 0.001) compared to the group that did not require new hospitalization in the period. Multivariate logistic regression showed that diabetes mellitus (OR 7.769; p < 0.001) and AKI (OR 7.995; p = 0.016) were independent risk factors for hospital readmission. There was no independent risk factor for short-term mortality in this cohort.

## DISCUSSION

In this study, IE affected patients with more comorbidities and older age. Most cases were healthcare-associated (62.3%), especially non-nosocomial (40.7%). *Staphylococcus* was the most common pathogen (59.8%). Patients who had serious complications during their hospital stay, such as stroke, septic shock and heart failure, had a less favorable prognosis (p < 0.001).

Although widely recognized, IE remains a challenge for healthcare systems in the current era due to its high morbidity, mortality and increasing incidence, particularly in older adults^(1,2)^. It is estimated that, in 2019, there were around 1.1 million cases of IE, with approximately sixty-six thousand deaths worldwide. With distinct characteristics in different regions of the world, the results of this study seek to elucidate some gaps regarding changes in its epidemiology in Brazil, a country that currently has one of the highest global incidence and mortality rates due to IE^(3)^.

The clinical profile of patients of this study was similar to that recently published in a systematic review on IE in Latin America: greater incidence in men, patients with valvular and ischemic heart diseases, other comorbidities such as hypertension and diabetes. Our results show a lower relevance of rheumatic valve disease as a predisposing factor and, as in high-income countries, patients with a higher mean age, mainly related to the death outcome^(20,21)^.

HAIE accounted for 62.3% of cases in this cohort, with 40.7% classified as non-nosocomial HAIE and 21.6% as nosocomial. Two national studies found similar results^(4,5)^, while European and Asian studies report a lower prevalence of HAIE, but with great variability in results^(19,22)^. The role of non-nosocomial acquisition of IE stands out, mostly related to previous hospital admission and procedures involving vascular access handling^(23)^. Although there were no differences in outcomes, it is worth noting that around 40% of patients in the cohort were on hemodialysis and the majority had an intravascular catheter device. In results from our previously published research, hemodialysis and recent hospital admission were independent risk factors for non-nosocomial HAIE^(11)^. It is known that this group has a higher prevalence of IE than the general population, mainly related to early episodes of bacteremia, especially due to *Staphylococcus aureus* ^(24)^, which may explain the high percentage of non-nosocomial HAIE cases in this cohort. Nurses have an important role to play in the prevention and identification of patients at high risk of HAIE, as they are the professionals with the most direct contact with patients and are responsible for ensuring that healthcare-associated infection precautions are effectively implemented^(25)^.

While in Latin America transesophageal echocardiograms are performed in around 60% of cases^(20)^, in this study it was performed on all patients. The mitral valve was the most affected, especially in the death outcome, followed by the aortic valve. Patients with death outcome had more complications associated with higher mortality, such as leaflet perforation and valve abscess^(21)^. They also presented a greater number of vegetations >10 mm, a risk factor for cerebral embolism when in the mitral valve and indicative of early surgical intervention^(21,26)^, which could justify a greater number of patients with embolic events in this group.

Approximately 40% of patients had a surgical indication; however, nearly 15% of patients with a death outcome did not undergo surgery despite the indication. Surgery was performed on average 22 days after diagnosis of IE, and this time remained unchanged over the study period. Although this was longer than recommended by international guidelines^(2,10)^, there was no difference in the outcomes analyzed. For patients in this cohort, failure to undergo surgical treatment in indicated cases seems to have played a more significant role in the unfavorable outcome than the time to surgery perform.

In relation to the microbiological profile, *Staphylococcus aureus* accounted for most cases of IE, a profile close to that found in high-income countries and Latin America^(20,27)^. CoNS also played a significant role in cases of this cohort, surpassing *Streptococcus* and *Enterococcus* in the number of infections. More than 80% of CoNS samples and 26% of *Staphylococcus aureus* (MRSA) samples showed methicillin resistance. Concerning resistance to other classes of antimicrobial agents, a considerable number of MDR and XDR samples also draw attention, especially in CoNS samples. The emergence of CoNS is reported in patients using intravascular devices, and like MRSA, it has a strong association with biofilm formation on these devices, making treatment more difficult and prolonged. Negative blood cultures were observed in 18% of the samples, a lower percentage than that found in Latin American studies and similar studies in high-income countries^(10,20)^. This percentage may be explained by the large number of patients (57%) using antimicrobial agents at culture collection as well as economic limitations or restricted access to serological tests and molecular biology techniques. Although this cohort did not find differences between the results, data from the EURO-ENDO registry show a possible increase in short-term mortality in patients with negative blood cultures and higher long-term mortality^(28)^.

As expected, patients of the death outcome group had more severe complications during hospital admission. Almost all these patients required admission to an ICU, and more than 80% required invasive ventilatory support. They also presented higher grades on the APACHE II and SOFA severity scores, useful tools for predicting mortality, demonstrating that some of the patients with this outcome already had more complex conditions when admitted to the ICU^(13)^.

It is worth highlighting the role of other healthcare-associated infections, mainly bloodstream infection and pneumonia, in the unfavorable outcome of these patients. More than 60% of patients who died developed septic shock *versus* 15% of patients who survived. In a Spanish multicenter study, infections caused by *Staphylococcus aureus* and gram-negative bacilli, hospital-acquired infections, persistent bacteremia, AKI, large vegetations and CNS embolization were predictive factors of septic shock in patients with IE. Septic shock has also been associated with several complications of IE, such as multiple organ involvement and worsening of previous or starting a new heart failure^(29)^. Almost half of patients with the death outcome presented AKI, large vegetations and embolic events, especially stroke, factors that may have contributed to the development of septic shock and the consequent worsening of these patients’ health conditions.

Hospital mortality was higher than that reported in Latin American studies^(20)^ and in high-income countries, but similar to other national studies^(5,6,9)^, placing Brazil as one of the countries with a higher risk of death from IE^(3)^. Short-term mortality was also high (51%), but there is no national data for comparison. There appears to be a tendency for change in the epidemiological profile of IE in the country, where healthcare-associated infections take the lead as the main sources of IE acquisition, especially in patients with vascular devices and history of bloodstream infection. It seems clear that now IE affects patients with more comorbidities, such as diabetes and those undergoing hemodialysis.

Short-term mortality in this cohort was higher in patients with comorbidities, with diabetes mellitus and AKI being independent risk factors for hospital readmission. Therefore, measures to prevent bacteremia, with glycemic control, skin care, strict care of central catheters, healthcare professional training in insertion and handling of intravascular devices, and the early identification of infections, are doable measures to reduce IE rates in this population^(23,24)^. In this context, nurses make significant contributions in both preventing new cases and managing complications. They are integral to all stages of care, working across different levels of care and complexity. With the increasing incidence of HAIE, nurses play a key role in intravascular catheters management, infection prevention, and health education for patients and the multidisciplinary team^(30)^.

The present study has limitations as it was conducted at a single center, with retrospective data analysis based on medical records, which can lead to loss of information. Follow-up was relatively short (six months) with few data available for analysis. Long-term follow-up data, two or more years, were not assessed. Although the results were similar to national prospective studies, all of them were carried out in southeastern Brazil and may not reflect the real situation in other areas of the country, making it necessary to carry out national multicenter studies.

## CONCLUSION

Short-term mortality was high (51%). Healthcare-associated infections were predominant in this cohort and affected older patients with associated comorbidities, consequently more vulnerable. Most infections were caused by *Staphylococcus aureus* and CoNS, with a significant number of MDR strains, especially in CoNS infections. This new scenario challenges healthcare services and requires greater involvement of the multiprofessional team in the early identification of patients at higher risk of IE, and mainly adherence to healthcare-associated infection prevention measures.

## Data Availability

The entire dataset supporting the results of this study is available on request from the corresponding author [Juliana Barros Becker]. The dataset is not publicly available as it contains information that compromises the privacy of the participants.
