# Supplementary material for: Clinical aspects and short-term prognosis in a cohort of patients with infective endocarditis, São Paulo, Brazil
Source: Rev Esc Enferm USP. 2025 Jul 28;59:e20250060. doi: 10.1590/1980-220X-REEUSP-2025-0060en (PMC12313321; doi:10.1590/1980-220X-REEUSP-2025-0060en)
Supplement: Translation

# Translation: Aspectos clínicos y pronóstico a corto plazo en cohorte de pacientes con endocarditis infecciosa, São Paulo, Brasil

**DOI:** 10.1590/1980-220X-REEUSP-2025-0060es

**Authors:** Juliana Barros Becker, Valdir Ambrósio Moisés, Dulce Aparecida Barbosa

**Affiliations:** 1 Universidade Federal de São Paulo, Hospital Universitário, São Paulo, SP, Brasil.; 2 Universidade Federal de São Paulo, Escola Paulista de Medicina, São Paulo, SP, Brasil.; 3 Universidade Federal de São Paulo, Escola Paulista de Enfermagem, Departamento de Enfermagem Clínica e Cirúrgica, São Paulo, SP, Brasil.

**Keywords:** Endocarditis, Infección Hospitalaria, Epidemiologia, Mortalidad Hospitalaria, Staphylococcus, Staphylococcus aureus Resistente a Meticilina

## Abstract

**Objetivo:**

Analizar las características clínicas de los pacientes con endocarditis infecciosa (EI) ingresados en un hospital terciario de Brasil, la mortalidad intrahospitalaria, los predictores de reingreso y la mortalidad hasta seis meses después del alta.

**Método:**

Estudio de cohorte retrospectivo, con recolección de datos de los registros médicos de los pacientes con diagnóstico de EI hospitalizados durante el período de estudio. Para el análisis estadístico comparativo, los pacientes se agruparon según los resultados de supervivencia y muerte.

**Resultados:**

204 pacientes participaron en el estudio. La EI asociada a la asistencia sanitaria representó el 62,3% de los casos, y el Staphylococcus aureus fue el patógeno predominante. La mortalidad se asoció significativamente a complicaciones como la insuficiencia cardiaca y el shock séptico (p < 0,001). La diabetes mellitus (OR 7,76; p < 0,001) y la lesión renal aguda (OR 7,99; p = 0,016) fueron factores de riesgo independientes de reingreso hospitalario. La mortalidad total fue del 50,9%.

**Conclusión:**

La mortalidad a corto plazo fue elevada. Predominaron las infecciones asociadas a la asistencia sanitaria, y las comorbilidades y complicaciones afectaron significativamente a la mortalidad de los pacientes con EI. La identificación de los pacientes de alto riesgo y la optimización de la gestión de cuidados pueden mejorar los resultados.

## INTRODUCCIÓN

La endocarditis infecciosa (EI) se define como una infección de la superficie endocárdica del corazón, de una válvula cardiaca nativa o protésica o de un dispositivo cardiaco permanente. Se trata de una enfermedad de magnitud mundial que cursa con una elevada mortalidad, cercana al 30% en un año, y una morbilidad significativa, que incluye la prolongación de la estancia hospitalaria, la reducción de la calidad de vida y un elevado riesgo de reinfección. A pesar de los avances, las tasas de incidencia han aumentado en los últimos años. En los países de ingresos altos, este aumento puede explicarse por cambios en el perfil epidemiológico, como el envejecimiento de la población, la mayor exposición a procedimientos sanitarios, el aumento del uso de dispositivos intracardiacos y vasculares y la aparición de infecciones por *Staphylococcus* ^(1,2)^.

En relación con América Latina, los datos del «Global Burden of Disease Study» indican que la región presentó las mayores tasas de incidencia de EI, y Brasil fue el país con el mayor aumento global en las tasas de incidencia de EI en 2019^(3)^. Estudios locales recientes muestran una mortalidad intrahospitalaria de entre el 32% y el 50%^(4,5,6,7,8,9)^, lo que sitúa a Brasil como uno de los países con mayor riesgo de muerte por EI.

La endocarditis infecciosa asociada a la asistencia a la salud (EIAS) ocupa actualmente un lugar destacado en la epidemiología de la enfermedad, representando entre el 25% y el 35% de los casos de EI en cohortes contemporáneas de países de renta alta y entre el 35% y el 67% de los casos en cohortes nacionales^(2,4,6)^. La más reciente guía sobre el manejo de la EI publicada por la Sociedad Europea de Cardiología enfatiza la importancia de la actuación integrada del equipo multidisciplinar en las medidas de prevención y educación sanitaria de los pacientes con mayor riesgo de EI^(10)^.

El escenario es desafiante y, a pesar de los datos alarmantes, los cambios en el perfil epidemiológico y microbiológico de los pacientes con EI en Brasil aún son poco conocidos. Algunos datos de la investigación, como las diferencias según la clasificación de la EI y los predictores de mortalidad intrahospitalaria, fueron publicados recientemente^(11)^. El presente estudio tuvo como objetivo analizar las características clínicas de los pacientes con EI ingresados en un hospital terciario de Brasil, la mortalidad intrahospitalaria y los predictores de reingreso y mortalidad hasta seis meses después del alta hospitalaria.

## MÉTODO

### Diseño y Contexto del Estudio

Se realizó un estudio de cohortes retrospectivo entre 2020 y 2023, con un período de recolección de datos de enero de 2009 a diciembre de 2019, en el *Hospital São Paulo*, *Universidade Federal de São Paulo*, Brasil. El hospital es una referencia en atención, docencia e investigación en Brasil.

### Participantes

Los pacientes con más de 18 años hospitalizados en el período analizado con sospecha de EI fueron seleccionados inicialmente a partir de la base de datos del laboratorio de ecocardiografía. Los pacientes fueron finalmente incluidos en el estudio tras el diagnóstico de EI confirmada o posible según los criterios de Duke modificados^(12)^ y si todos los datos estaban disponibles en las historias clínicas físicas (2009 a 2015) y electrónicas (2016 a 2019). Los pacientes se dividieron y compararon en dos grupos según los resultados de supervivencia o muerte. Se analizaron los pacientes que sobrevivieron y continuaron el seguimiento, incluidos los reingresos hospitalarios y las muertes dentro de los seis meses posteriores al alta.

### Recogida de Datos

Los datos recolectados incluyeron características demográficas, condición cardiaca predisponente para la EI, comorbilidades, microorganismos causantes, modo de adquirir la EI, hallazgos clínicos y ecocardiográficos, complicaciones y tratamiento quirúrgico. Para los pacientes ingresados en la Unidad de Cuidados Intensivos (UCI) durante más de 48 horas, se calcularon las puntuaciones Acute Physiology and Chronic Health Evaluation (APACHE II) y Sequential Organ Failure Assessment (SOFA) en las primeras 24 horas^(13,14)^.

### Definiciones de las Variables

Se definieron como cardiopatías predisponentes los antecedentes de sustitución valvular cardiaca, defectos cardiacos congénitos, valvulopatías, episodios previos de EI e implantación de dispositivos cardiacos como marcapasos o desfibrilador. Las complicaciones de la EI incluyeron la insuficiencia cardiaca aguda, el shock cardiogénico, las embolias arteriales, incluido el infarto pulmonar, la sepsis, el shock séptico y la lesión renal aguda (LRA) que requirió hemodiálisis. El shock cardiogénico se definió según los estadios C, D y E de la Society for Cardiovascular Angiography and Intervention^(15)^. La sepsis se definió como una disfunción orgánica causada por una respuesta desregulada a la infección. El shock séptico se definió como sepsis con hipotensión resistente que requiere vasopresores para mantener una presión arterial media ≥ 65 mmHg^(16)^. La LRA se definió de acuerdo con la clasificación Kidney Disease: Improving Global Outcomes (KDIGO) criteria^(17)^. Los agentes causales de la EI se dividieron según el patrón de resistencia en susceptibles, multirresistentes (MDR), extremadamente resistentes (XDR) y pan-drogo-resistentes (PDR). La MDR se definió como la no susceptibilidad adquirida frente a al menos un agente de tres o más categorías de antimicrobianos; la XDR se definió como la no susceptibilidad frente a al menos un agente de todas las categorías de antimicrobianos excepto dos o menos; y la PDR se definió como la no susceptibilidad frente a todos los agentes de todas las categorías de antimicrobianos^(18)^.

Los modos de adquisición de la EI fueron EIAS, o la endocarditis infecciosa adquirida en la comunidad (EIC). La EIAS se dividió en no nosocomial y nosocomial. La EI no nosocomial se definió como la ocurrida antes o en las 48 horas siguientes al ingreso hospitalario en un paciente con amplia exposición extrahospitalaria a intervenciones médicas; ingreso hospitalario durante dos o más días en los 90 días anteriores al inicio de la EI; residencia en una residencia de ancianos o centro de cuidados de larga duración antes del ingreso hospitalario. Las EI nosocomiales se definieron como aquellas cuyos signos y síntomas compatibles con EI se desarrollaron después de 48 horas o más del ingreso hospitalario. La EI se definió como aquella cuyos signos y síntomas se desarrollaron antes o en las 48 horas siguientes al ingreso hospitalario y que no cumplía los criterios de EI no nosocomial^(19)^.

### Análisis Estadístico

Los datos recolectados fueron analizados utilizando el software libre R versión 4.2.2 para Windows. En el análisis descriptivo de los datos, las variables cuantitativas se resumen mediante media, desviación estándar, mediana, rango intercuartílico según el tipo de distribución; las variables cualitativas se presentan como números y porcentajes. Asimismo, se realizaron análisis estadísticos comparativos entre grupos mediante la prueba de chi-cuadrado o la prueba exacta de Fisher para las variables cualitativas. Para las variables cuantitativas, se utilizó la prueba t de Student o la prueba de Mann-Whitney. Se realizó una regresión logística multivariante para identificar las variables relacionadas con el reingreso hospitalario y la mortalidad tras el alta, y se calculó la odds ratio con un intervalo de confianza del 95%. Los valores p inferiores a 0,05 se consideraron estadísticamente significativos.

### Aspectos Éticos

El estudio fue aprobado por el Comité de Ética en Investigación de *la Universidad Federal de São Paulo* (Resolución 673/2019, Dictamen 3.588.513).

## RESULTADOS

La muestra inicial consistió en 278 pacientes con sospecha de EI; de ellos, 204 cumplieron los criterios de inclusión. En la Tabla 1 se resumen las principales características clínicas. Los hombres representaban el 57,8% de esta cohorte, sin diferencias entre los resultados. La edad media fue de 53 años, con una media superior en los pacientes en el desenlace de muerte (p < 0,001). Se observó un mayor porcentaje de antecedentes de cardiopatía isquémica (p = 0,003) e hipertensión (p = 0,026) en el desenlace de muerte. En cuanto a la supervivencia, se observó un mayor porcentaje de pacientes con antecedentes de infección reciente del torrente sanguíneo (hasta 90 días) (p = 0,019). En cuanto a los síntomas, se observó fiebre en el 88,7% de los pacientes y soplo cardiaco en el 52,5%, sin diferencias entre los grupos.

**Tabla 1:** Características de los pacientes con endocarditis infecciosa según los resultados – São Paulo, SP, Brasil, 2009–2019.

	Supervivencia (n = 113)	Muerte (n = 91)	Total (n = 204)	p-valor
Hombre	64 (56.6)	54 (59.3)	118 (57.8)	0.697
Edad mediana (DE)	47.45 (16.779)	60.11 (14.373)	53.21 (16.887)	<0.001
Condiciones cardíacas predisponentes				
Enfermedad valvular previa	18 (15.9)	23 (25.3)	41 (20)	0.097
Cardiopatía reumática	11 (9.7)	8 (8.8)	19 (9.3)	0.817
Episodio anterior de EI	10 (8.8)	9 (9.9)	19 (9.3)	0.799
Dispositivo cardíaco	6 (5.3)	8 (8.8)	14 (6.9)	0.328
Cirugía cardíaca previa				
Cirugía de revascularización oronaria	2 (1.8)	5 (5.5)	7 (3.4)	0.246
Cirugía valvular	14 (12.4)	16 (17.6)	30 (14.7)	0.298
Otros	8 (7)	6 (6.6)	14 (6.9)	0.891
Comorbilidades				
Cardiopatía isquémica	10 (8.8)	22 (24.2)	32 (15.7)	0.003
Insuficiencia cardiaca	23 (20.4)	21 (23)	44 (21.6)	0.638
Otras cardiopatías	18 (15.9)	18 (19.8)	36 (17.6)	0.473
Hipertensión arterial	65 (57.5)	66 (72.5)	131 (64.2)	0.026
Cáncer	14 (12.4)	12 (13.2)	27 (13.2)	0.817
Diabetes	28 (24.8)	28 (30.7)	56 (27.5)	0.341
Hemodiálisis	42 (37.2)	35 (38.5)	77 (37.7)	0.847
Catéter intravascular (central)	39 (34.5)	27 (29.7)	66 (32.4)	0.462
Infección reciente del torrente sanguíneo	14 (12.4)	3 (3.3)	17 (8.3)	0.019
Ingreso hospitalario reciente	30 (26.5)	27 (29.7)	57 (27.9)	0.621
Endocarditis de válvula protésica	12 (10.6)	14 (15.4)	26 (12.7)	0.310
CIE	43 (38)	34 (37.4)	77 (37.7)	0.919
EIAS no nosocomial	51 (45.1)	32 (35.2)	83 (40.7)	0.149
EIAS nosocomial	19 (16.8)	25 (27.5)	44 (21.6)	0.066
Hallazgos clínicos en el momento de la presentación				
Fiebre	102 (90.3)	79 (86.8)	181 (88.7)	0.438
Soplo cardíaco	56 (49.6)	51 (56)	107 (52.5)	0.356
Criterios de Duke				
Mayor				
Ecocardiograma positivo	84 (74.3)	87 (95.6)	171 (83.8)	<0.001
Hemocultivo positivo	47 (41.6)	43 (47.3)	90 (44.2)	0.418
Menor				
Cardiopatía predisponente	41 (36.3)	45 (49.5)	86 (42.2)	0.058
Uso de catéter venoso central	44 (38.9)	21 (23)	65 (31.8)	0.016
Fenómenos vasculares	41 (36.3)	44 (48.4)	85 (41.7)	0.082
Fenómenos inmunológicos	19 (16.8)	18 (19.8)	37 (18.2)	0.585
Hemocultivo (no mayor)	25 (22.2)	3 (3.3)	28 (13.7)	<0.001
EI				
Posible	41 (36.3)	12 (13.2)	53 (26)	<0.001
Definitivo	72 (63.7)	79 (86.8)	151 (74)	<0.001

Nota: Valor p = prueba de chi cuadrado o prueba exacta de Fisher para variables cualitativas y prueba de la t de Student para variables cuantitativas; DE: desviación estándar; EIC = endocarditis infecciosa adquirida en la comunidad; EIAS = endocarditis infecciosa asociada a la asistencia de salud; EI = endocarditis infecciosa.

Se diagnosticó EI definitiva en 151 pacientes (74%), con una proporción significativamente mayor en el grupo de muerte (p < 0,0001), y posible EI en 53 pacientes (26%), con una proporción mayor en el grupo de supervivencia. La EIAS representó el 62,3% de los casos de esta cohorte; el 40,7% se clasificaron como no nosocomiales y el 21,6% como nosocomiales, sin diferencias significativas entre los grupos.

Se realizó ecocardiografía transesofágica a todos los pacientes. La válvula mitral fue la más afectada por la vegetación, especialmente en el desenlace de muerte (p = 0,026), seguida de la válvula aórtica, sin diferencias entre grupos. En el desenlace mortal se observó un mayor número de vegetaciones mayores de 10 mm (p = 0,003) y de complicaciones, como perforación de valvas, pseudoaneurisma y absceso perivalvular (p = 0,016).

Los hemocultivos fueron negativos en el 18,1% de los casos, sin diferencias significativas entre los grupos. Entre los hemocultivos positivos, se encontraron bacterias grampositivas en el 77% de los casos, de las cuales el 45,9% eran *Staphylococcus aureus* y el 31,8% *Staphylococcus* coagulasa-negativos (CoNS). Alrededor del 26% de las muestras de *Staphylococcus aureus* y el 84% de las de CoNS mostraron resistencia a la meticilina, sin diferencias significativas entre los grupos. Se observó resistencia clasificada como MDR en el 35% y como XDR en el 5% (Figura 1). Se realizaron cultivos de tejido endocárdico o valvular en cuarenta pacientes (57,1%) sometidos a tratamiento quirúrgico. De éstos, el 85% no mostró crecimiento microbiano, sin diferencias entre los resultados. No hubo cambios en las directrices internacionales sobre el tratamiento antimicrobiano de la EI durante el periodo de estudio^(2)^. El tratamiento farmacológico utilizado fue de agentes antimicrobianos b-lactámicos en el 91%, y de glucopéptidos en el 84%, sin diferencias significativas entre los grupos. Entre los pacientes con resultado de muerte, hubo un mayor uso de aminoglucósidos (p = 0,027), polipéptidos (p < 0,001) y antifúngicos (p = 0,004) durante la estancia hospitalaria.

**Figura 1:** Agentes causantes de endocarditis infecciosa según el perfil de resistência.

Nota: MDR – Multi-drogo resistente; XDR – Extremadamente drogo-resistente.

Los pacientes con resultado de muerte presentaron la mayoría de las complicaciones. Se produjeron eventos embólicos en alrededor del 60% de los pacientes de este grupo (p = 0,002), y el sistema nervioso central fue el más afectado (p < 0,001). En el 47% de estos pacientes se observó una LRA que requirió hemodiálisis (p < 0,001). Las infecciones relacionadas con la asistencia sanitaria también se observaron con mayor frecuencia en los pacientes con este resultado: El 57,1% contrajo infección del torrente sanguíneo y el 58,2% neumonía (p < 0,001). Más del 60% de los pacientes con desenlace mortal desarrollaron shock séptico (p < 0,001); el 48%, insuficiencia cardiaca (p < 0,001); el 25%, shock cardiogénico (p < 0,001); el 29,7%, parada cardiorrespiratoria (p<0,001); el 83%, ventilación mecánica invasiva (p < 0,001); y el 94,5%, ingreso en UCI (p < 0,001). Los pacientes con resultado de muerte también tenían puntuaciones más altas en APACHE II (p = 0,001) y SOFA (p = 0,002). La duración media de la estadía hospitalaria fue de 44,67 días, con una media superior para la supervivencia (p = 0,016) (Tabla 2).

**Tabla 2:** Complicaciones observadas durante la internación hospitalaria de pacientes con endocarditis infecciosa según desenlaces – São Paulo, SP, Brasil, 2009–2019.

	Supervivencia (n = 113)	Muerte (n = 91)	Total (n = 204)	p-valor
Embolias	41 (36.3)	53 (58.2)	94 (46.1)	0.002
Sistema nervioso central	14 (12.4)	34 (37.4)	48 (23.5)	<0.001
Pulmonar	10 (8.8)	2 (2.2)	12 (5.9)	0.045
Osteoarticular	4 (3.5)	6 (6.6)	10 (4.9)	0.346
Otros	13 (11.5)	11 (12.1)	24 (11.8)	0.897
Complicaciones infecciosas				
Sepsis	28 (24.8)	22 (24.17)	50 (24.5)	0.921
Shock séptico	17 (15)	58 (63.7)	75 (36.8)	<0.001
EIAS				
Infección del torrente sanguíneo	36 (31.8)	52 (57.1)	88 (43.1)	<0.001
Neumonía	28 (24.8)	53 (58.2)	81 (39.7)	<0.001
Infección urinaria	15 (13.3)	18 (19.8)	33 (16.2)	0.209
Otros	14 (12.4)	6 (6.6)	20 (9.8)	0.166
Insuficiencia cardiaca	17 (15)	44 (48.4)	61 (29.9)	<0.001
Shock cardiogénico	1 (0.9)	23 (25.3)	24 (11.8)	<0.001
Parada cardiorrespiratoria	3 (2.7)	27 (29.7)	30 (14.7)	<0.001
LRA				
LRA sin hemodiálisis	28 (24.8)	17 (18.7)	45 (22.1)	0.296
LRA con hemodiálisis	15 (13.3)	43 (47.2)	58 (28.4)	<0.001
Transfusión de concentrado de hematíes	39 (34.5)	55 (60.4)	94 (46.1)	<0.001
Ventilación mecánica invasiva	32 (28.3)	76 (83.5)	108 (52.9)	<0.001
UCI	71 (62.8)	86 (94.5)	157 (76.9)	<0.001
Severidad: Puntuaciones UCI ||	16.4 (9.9)	22.24 (8.75)	19.5 (9.72)	<0.001
Apache II (Media DE)	28 (40.6)	7 (8.8)	35 (23.5)	<0.001
0 a 14 puntos (15% de riesgo de muerte)	37 (53.6)	55 (68.8)	92 (61.7)	0.058
15 a 29 puntos (25–55% de riesgo de muerte)	4 (5.8)	18 (22.5)	22 (14.8)	0.004
30 a > 34 puntos (73–85% de riesgo de muerte)	6.61 (3.61)	8.419 (3.998)	7.591 (3.992)	0.002
SOFA	28 (40.6)	15 (18.3)	43 (28.5)	0.002
0 a 5 puntos (20,2% de riesgo de muerte)	37 (53.6)	51 (62.2)	88 (58.2)	0.287
De 6 a 11 puntos (50% de riesgo de muerte)	4 (5.8)	16 (19.5)	20 (13.2)	0.013
>12 puntos (95,2% de riesgo de muerte)	14.45 (13.416)	22.5 (25.053)	18 (20.877)	0.075
Duración de la estadía en la UCI	45.5 (21.57)	43.65 (36.3)	44.67 (29.03)	0.016

Nota: EIAS: Endocarditis infecciosa relacionada con la asistencia a la salud. LRA: lesión renal aguda; UCI: unidad de cuidados intensivos; p-valor = prueba de chi cuadrado o prueba exacta de Fisher para variables cualitativas y prueba de Mann-Whitney para variables cuantitativas; ^||^Puntuaciones de gravedad en la UCI: calculadas en 149 pacientes; APACHE II = puntuación en la evaluación de la fisiología aguda y la salud crónica; DE = desviación estándar; SOFA = puntuación en la evaluación secuencial del fallo orgánico.

El 42,6% de los pacientes de esta cohorte tenían indicación quirúrgica, y el 34,3% de ellos fueron sometidos a tratamiento quirúrgico. De los pacientes que tenían indicación quirúrgica, casi el 20% no se sometieron a cirugía, principalmente debido al resultado de muerte (p = 0,024). El tiempo medio transcurrido entre el diagnóstico de la EI y la intervención quirúrgica fue de 22 días. La cirugía de sustitución valvular con colocación de prótesis biológica se realizó en el 56% de los casos. Las válvulas mitral (44,3%) y aórtica (21,4%) fueron las más afectadas, sin diferencias entre los resultados.

De los 204 pacientes de esta cohorte, 91 (44,6%) fallecieron durante la estancia hospitalaria. De los 113 pacientes que sobrevivieron, 82 (72,6%) mantuvieron el seguimiento en la institución en los seis meses siguientes al alta. De ellos, 30 pacientes (36,6%) requirieron un nuevo ingreso hospitalario en este periodo, y 13 pacientes más fallecieron. Así, desde el primer ingreso hospitalario y considerando los pacientes en seguimiento, fallecieron un total de 104 pacientes.

Las principales causas de reingreso hospitalario fueron la sepsis (56,7%) y la insuficiencia cardiaca (20%). La infección del torrente sanguíneo y la neumonía fueron las principales causas de infección. La recurrencia de EI supuso el 10% de los casos. Los pacientes que requirieron un nuevo ingreso hospitalario tenían una media de edad más elevada (50 años; p = 0,04) y un mayor porcentaje de hipertensión (76,6%) (p = 0,003), diabetes mellitus (46,7%) y hemodiálisis (63,3%) (p < 0,001) en comparación con el grupo que no requirió un nuevo ingreso hospitalario en el periodo. La regresión logística multivariante mostró que la diabetes mellitus (OR 7,769; p < 0,001) y la LRA (OR 7,995; p = 0,016) eran factores de riesgo independientes para el reingreso hospitalario. No hubo ningún factor de riesgo independiente de mortalidad a corto plazo en esta cohorte.

## DISCUSIÓN

En este estudio, la EI afectó a pacientes con más comorbilidades y mayor edad. La mayoría de los casos fueron asociados a la asistencia sanitaria (62,3%), especialmente no nosocomiales (40,7%). El *Staphylococcus (estafilococo)* fue el patógeno más frecuente (59,8%). Los pacientes que presentaron complicaciones graves durante su estancia hospitalaria, como ictus, shock séptico e insuficiencia cardiaca, tuvieron un pronóstico menos favorable (p < 0,001).

Aunque ampliamente reconocida, la EI sigue siendo un reto para los sistemas sanitarios en la era actual debido a su elevada morbilidad, mortalidad y creciente incidencia, sobre todo en adultos mayores^(1,2)^. Se estima que, en 2019, hubo alrededor de 1,1 millones de casos de EI, con aproximadamente sesenta y seis mil muertes en todo el mundo. Con características distintas en diferentes regiones del mundo, los resultados de este estudio buscan dilucidar algunas lagunas en relación a los cambios en su epidemiología en Brasil, país que actualmente presenta una de las mayores tasas mundiales de incidencia y mortalidad por EI^(3)^.

El perfil clínico de los pacientes de este estudio fue similar al publicado recientemente en una revisión sistemática sobre EI en Latinoamérica: mayor incidencia en hombres, pacientes con cardiopatías valvulares e isquémicas, otras comorbilidades como hipertensión y diabetes. Nuestros resultados muestran una menor relevancia de la valvulopatía reumática como factor predisponente y, al igual que en los países de altos ingresos, pacientes con una media de edad más elevada, relacionada principalmente con el desenlace de muerte^(20,21)^.

La EIAS representó el 62,3% de los casos de esta cohorte, con un 40,7% clasificado como EIAS no nosocomial y un 21,6% como nosocomial. Dos estudios nacionales encontraron resultados similares^(4,5)^, mientras que estudios europeos y asiáticos informan de una menor prevalencia de EIAS, pero con gran variabilidad en los resultados^(19,22)^. Destaca el papel de la adquisición no nosocomial de EI, en su mayoría relacionada con ingresos hospitalarios previos y procedimientos que implican la manipulación de accesos vasculares^(23)^. Aunque no hubo diferencias en los resultados, cabe destacar que alrededor del 40% de los pacientes de la cohorte estaban en hemodiálisis y la mayoría disponía de un dispositivo de catéter intravascular. En los resultados de nuestra investigación publicada anteriormente, la hemodiálisis y el ingreso hospitalario reciente fueron factores de riesgo independientes de la EIAS no nosocomial^(11)^. Se sabe que este grupo tiene una mayor prevalencia de EI que la población general, principalmente relacionada con episodios tempranos de bacteriemia, sobre todo debida a *Staphylococcus aureus* ^(24)^, lo que puede explicar el alto porcentaje de casos de EIAS no nosocomial en esta cohorte. El personal de enfermería tiene un importante papel que desempeñar en la prevención y la identificación de los pacientes con alto riesgo de IRAS, ya que son los profesionales que tienen un contacto más directo con los pacientes y son los responsables de garantizar que se apliquen eficazmente las precauciones frente a las infecciones relacionadas con la asistencia sanitaria^(25)^.

Mientras que en Latinoamérica el ecocardiograma transesofágico se realiza en alrededor del 60% de los casos^(20)^, en este estudio se realizó en todos los pacientes. La válvula mitral fue la más afectada, especialmente en el desenlace mortal, seguida de la válvula aórtica. Los pacientes con desenlace mortal presentaron más complicaciones asociadas a mayor mortalidad, como perforación de valvas y absceso valvular^(21)^. También presentaban un mayor número de vegetaciones >10 mm, factor de riesgo de embolia cerebral en la válvula mitral e indicativo de intervención quirúrgica precoz^(21,26)^, lo que podría justificar un mayor número de pacientes con eventos embólicos en este grupo.

Aproximadamente el 40% de los pacientes tenían una indicación quirúrgica; sin embargo, casi el 15% de los pacientes con resultado de muerte no se sometieron a cirugía a pesar de la indicación. La cirugía se realizó una media de 22 días después del diagnóstico de EI, y este tiempo se mantuvo invariable durante el periodo de estudio. Aunque este tiempo fue superior al recomendado por las directrices internacionales^(2,10)^, no hubo diferencias en los resultados analizados. En los pacientes de esta cohorte, el hecho de no someterse a tratamiento quirúrgico en los casos indicados parece haber desempeñado un papel más importante en el resultado desfavorable que el tiempo transcurrido hasta la intervención quirúrgica.

En relación con el perfil microbiológico, *Staphylococcus aureus* representó la mayoría de los casos de EI, un perfil cercano al encontrado en países de renta alta y Latinoamérica^(20,27)^. Los CoNS también desempeñaron un papel importante en los casos de esta cohorte, superando a *Streptococcus* y *Enterococcus* en el número de infecciones. Más del 80% de las muestras de CoNS y el 26% de las de *Staphylococcus aureus* (SARM) mostraron resistencia a la meticilina. En cuanto a la resistencia a otras clases de agentes antimicrobianos, también llama la atención un número considerable de muestras MDR y XDR, especialmente en las muestras de CoNS. Se ha descrito la aparición de CoNS en pacientes que utilizan dispositivos intravasculares y, al igual que el SARM, tiene una fuerte asociación con la formación de biopelículas en estos dispositivos, lo que dificulta y prolonga el tratamiento. Se observaron hemocultivos negativos en el 18% de las muestras, un porcentaje inferior al encontrado en estudios latinoamericanos y en estudios similares en países de ingresos altos^(10,20)^. Este porcentaje puede explicarse por el gran número de pacientes (57%) que utilizaban agentes antimicrobianos en el momento de recolectar los cultivos, así como por las limitaciones económicas o el acceso restringido a las pruebas serológicas y las técnicas de biología molecular. Aunque en esta cohorte no se encontraron diferencias entre los resultados, los datos del registro EURO-ENDO muestran un posible aumento de la mortalidad a corto plazo en los pacientes con hemocultivos negativos y una mayor mortalidad a largo plazo^(28)^.

Como era de esperar, los pacientes del grupo con resultado de muerte presentaron complicaciones más graves durante el ingreso hospitalario. Casi todos estos pacientes precisaron ingreso en una UCI, y más del 80% requirieron asistencia ventilatoria invasiva. También presentaron grados más altos en las puntuaciones de gravedad APACHE II y SOFA, herramientas útiles para predecir la mortalidad, lo que demuestra que algunos de los pacientes con este desenlace ya presentaban afecciones más complejas cuando ingresaron en la UCI^(13)^.

Cabe destacar el papel de otras infecciones relacionadas con la asistencia sanitaria, principalmente la infección del torrente sanguíneo y la neumonía, en el resultado desfavorable de estos pacientes. Más del 60% de los pacientes que fallecieron desarrollaron un shock séptico *frente al* 15% de los pacientes que sobrevivieron. En un estudio multicéntrico español, las infecciones causadas por *Staphylococcus aureus* y bacilos gramnegativos, las infecciones adquiridas en el hospital, la bacteriemia persistente, la LRA, las vegetaciones grandes y la embolización del SNC fueron factores predictivos de shock séptico en pacientes con EI. El shock séptico también se ha asociado a varias complicaciones de la EI, como la afectación de múltiples órganos y el empeoramiento de una insuficiencia cardiaca previa o el inicio de una nueva^(29)^. Casi la mitad de los pacientes con resultado de muerte presentaban LRA, grandes vegetaciones y eventos embólicos, especialmente ictus, factores que pueden haber contribuido al desarrollo del shock séptico y al consiguiente empeoramiento del estado de salud de estos pacientes.

La mortalidad hospitalaria fue mayor que la reportada en estudios latinoamericanos^(20)^ y en países de altos ingresos, pero similar a la de otros estudios nacionales^(5,6,9)^, ubicando a Brasil como uno de los países con mayor riesgo de muerte por EI^(3)^. La mortalidad a corto plazo también fue elevada (51%), pero no hay datos nacionales para la comparación. Parece haber una tendencia al cambio en el perfil epidemiológico de la EI en el Brasil, donde las infecciones asociadas a la asistencia sanitaria toman la delantera como principales fuentes de adquisición de EI, especialmente en pacientes con dispositivos vasculares y antecedentes de infección del torrente sanguíneo. Parece claro que ahora la EI afecta a pacientes con más comorbilidades, como la diabetes y los sometidos a hemodiálisis.

La mortalidad a corto plazo en esta cohorte fue mayor en los pacientes con comorbilidades, siendo la diabetes mellitus y la LRA factores de riesgo independientes de reingreso hospitalario. Por lo tanto, las medidas para prevenir la bacteriemia, con control glucémico, cuidado de la piel, cuidado estricto de los catéteres centrales, formación de los profesionales sanitarios en la inserción y manipulación de dispositivos intravasculares, y la identificación precoz de las infecciones, son medidas factibles para reducir las tasas de EI en esta población^(23,24)^. En este contexto, el personal de enfermería contribuye de forma significativa tanto a la prevención de nuevos casos como a la gestión de las complicaciones. Forman parte integrante de todas las fases de la asistencia y trabajan en distintos niveles de atención y complejidad. Con el aumento de la incidencia de la EIAS, las enfermeras desempeñan un papel clave en el manejo de los catéteres intravasculares, la prevención de infecciones y la educación sanitaria de los pacientes y el equipo multidisciplinar^(30)^.

El presente estudio tiene limitaciones, ya que se realizó en un único centro, con un análisis retrospectivo de los datos basado en las historias clínicas, lo que puede dar lugar a pérdidas de información. El seguimiento fue relativamente corto (seis meses) y se dispuso de pocos datos para el análisis. No se evaluaron los datos de seguimiento a largo plazo, dos o más años. Aunque los resultados fueron similares a los de los estudios prospectivos nacionales, todos ellos se llevaron a cabo en el sudeste de Brasil y pueden no reflejar la situación real en otras zonas del país, por lo que es necesario llevar a cabo estudios multicéntricos nacionales.

## CONCLUSIÓN

La mortalidad a corto plazo fue elevada (51%). Las infecciones relacionadas con la asistencia sanitaria predominaron en esta cohorte y afectaron a pacientes de edad avanzada con comorbilidades asociadas, en consecuencia, más vulnerables. La mayoría de las infecciones fueron causadas por *Staphylococcus aureus* y CoNS, con un número significativo de cepas MDR, especialmente en las infecciones por CoNS. Este nuevo escenario supone un reto para los servicios sanitarios y requiere una mayor implicación del equipo multiprofesional en la identificación precoz de los pacientes con mayor riesgo de EI y, principalmente, el cumplimiento de las medidas de prevención de las infecciones relacionadas con la asistencia sanitaria.

## Data Availability

El conjunto de datos completo que respalda los resultados de este estudio está disponible previa solicitud a la autora correspondiente [Juliana Barros Becker]. El conjunto de datos no está a disposición del público, ya que contiene información que pone en peligro la privacidad de los participantes.
